# Intracranial Aneurysm Segmentation with a Dual-Path Fusion Network

**DOI:** 10.3390/bioengineering12020185

**Published:** 2025-02-15

**Authors:** Ke Wang, Yong Zhang, Bin Fang

**Affiliations:** College of Computer Science, Chongqing University, Chongqing 400038, China; wangkecqu@163.com (K.W.); zhangyong7630@163.com (Y.Z.)

**Keywords:** intracranial aneurysms segmentation, digital subtraction angiography, dual-path, view fusion

## Abstract

Intracranial aneurysms (IAs), a significant medical concern due to their prevalence and life-threatening nature, pose challenges regarding diagnosis owing to their diminutive and variable morphology. There are currently challenges surrounding automating the segmentation of IAs, which is essential for diagnostic precision. Existing deep learning methods in IAs segmentation tend to emphasize semantic features at the expense of detailed information, potentially compromising segmentation quality. Our research introduces the innovative Dual-Path Fusion Network (DPF-Net), an advanced deep learning architecture crafted to refine IAs segmentation by adeptly incorporating detailed information. DPF-Net, with its unique resolution-preserving detail branch, ensures minimal loss of detail during feature extraction, while its cross-fusion module effectively promotes the connection of semantic information and finer detail features, enhancing segmentation precision. The network also integrates a detail aggregation module for effective fusion of multi-scale detail features. A view fusion strategy is employed to address spatial disruptions in patch generation, thereby improving feature extraction efficiency. Evaluated on the CADA dataset, DPF-Net achieves a remarkable mean Dice similarity coefficient (DSC) of 0.8967, highlighting its potential in automated IAs diagnosis in clinical settings. Furthermore, DPF-Net’s outstanding performance on the BraTS 2020 MRI dataset for brain tumor segmentation with a mean DSC of 0.8535 further confirms its robustness and generalizability.

## 1. Introduction

Intracranial aneurysms (IAs) are a common vascular anomaly characterized by local dilations in weak areas of the blood vessel walls [1,2]. Their high incidence and serious threat to life have led to considerable attention on management and treatment strategies [3,4]. Most IAs have a low risk of rupture, requiring only periodic monitoring [1]. However, in cases of rupture, the consequences can be profoundly severe, often leading to subarachnoid hemorrhage (SAH), a condition that demands immediate attention and poses a significant threat to life [5,6,7,8,9,10]. Therefore, the early detection of aneurysms is crucial for timely assessment and the implementation of appropriate treatment plans. Currently, the clinical delineation of IAs predominantly depends on manual methods conducted in a slice-by-slice manner, which is dull, time-consuming, and subjective. Therefore, there is an urgent need for the development of an automated IAs segmentation method for clinical application to reduce the workload on medical staff and ensure a consistent approach to the diagnosis and management of this critical health issue.

In the past decade, the robust development of deep learning algorithms, coupled with remarkable advancements in computational capabilities, has instigated a paradigm shift in medical image analysis. These advanced technologies have surpassed traditional methods and emerged as the cornerstone in this evolving field [11,12,13]. Their deployment has catalyzed key breakthroughs in various medical imaging tasks, notably in enhancing the precision of organ segmentation (e.g., the liver [14], airway [15], and pancreas [16]), improving the accuracy of lesion detection (e.g., pulmonary nodule [17,18]), and optimizing tumor segmentation (e.g., liver tumor [14,19], pancreas tumor [20], and brain tumor [12]). Despite these advancements, the accurate segmentation of IAs remains a challenging task. First, IAs constitute a very small portion of the total volume, typically less than 0.2%, which can result in under-segmentation or misdiagnosis due to the predominance of larger background structures. Second, IAs, being structural anomalies within arterial vessels, often mimic the density distribution of the surrounding vasculature, complicating their detection. Third, the significant variation in size, shape, and location of IAs poses challenges to achieving accurate automatic segmentation.

To enhance the segmentation performance of IAs, several deep learning-based approaches have been proposed [9,10,21,22,23,24,25,26]. Stember et al. [23] and Chen et al. [2] developed a U-Net framework for cerebral aneurysm segmentation. Chen et al. [27] introduced an active contour technique for discerning aneurysms in high-contrast areas. Park et al. [28] introduced the HeadXNet network in their cerebral aneurysms assisted diagnosis system to enhance physicians’ detection accuracy. Shi et al. [1] improved aneurysm detection by incorporating dual attention blocks into 3D CNNs, coupled with a sample-balancing strategy. Mu et al. [3] devised an attention residual U-Net with specialized pre- and post-processing to enhance segmentation integrity. Ou et al. [4] integrated a cross-scale dual-path transformer module into 3D U-Net to effectively merge local and global information to detect varied-sized aneurysms.

Despite these advancements, two major limitations persist. The first limitation is the loss of detailed information. The accuracy of semantic segmentation hinges on maintaining detail and semantic information. However, in aneurysm studies, the widely used single-path U-Net architecture often fails to maintain finer details during the down-sampling process. This limitation results in the loss of critical small-scale features, such as the subtle contours and shapes of smaller aneurysms. Consequently, this can lead to the oversight of smaller aneurysms and an increased risk of lesion misdetection. The second limitation is the insufficient utilization of spatial features. Although 3D CNNs effectively capture spatial features, the prevalent patch-based training approach can compromise the spatial integrity of targets. Specifically, this approach divides the target volume into smaller patches, which can disrupt the overall spatial coherence of the targets. As a result, important 3D spatial relationships, such as the full shape and depth of aneurysms, may not be fully captured during the training process. This often results in an inadequate extraction of the aneurysm’s 3D characteristics, increasing the risk of segmentation errors.

To address the aforementioned challenges, we propose an innovative end-to-end segmentation system. Compared to existing approaches, our proposed approach has three principal innovations. Firstly, a dual-path fusion network (DPF-Net) is specifically designed to effectively minimize detail loss in semantic feature extraction. Specifically, DPF-Net incorporates a detail branch that preserves fine-grained features by extracting contextual information without any down-sampling operations. In parallel, a semantic branch is used to capture deep semantic feature maps, allowing the network to maintain a balanced representation of both detailed and high-level semantic information. Additionally, a cross-fusion module is proposed to promote the information interaction between the detail branch and the semantic branch. Furthermore, a detail aggregation module is integrated into the detail branch for optimal detail feature utilization across various stages. Second, thanks to the detail branch’s ability to extract and preserve fine-grained features, we can easily achieve deep semantic feature restoration without the cumbersome layer-by-layer up-sampling used in other methods. This makes our approach more resource-efficient, with a parameter size of 1.45 MB, significantly smaller than the 4.38 MB of the classical 3D U-Net and 12.48 MB of the 3D Attention U-Net. Third, to promote the sufficient utilization of spatial features, we implement a view fusion technique in both training and testing, which simulates the manual delineation process by considering multiple perspectives (i.e., axial, coronal, and sagittal). Specifically, this technique generates patches from multiple perspectives, significantly mitigating spatial structure disruptions and preserving the integrity of the aneurysm’s 3D structure.

Compared to several state-of-the-art 3D IAs segmentation methods on the widely recognized publicly available IAs dataset (Cerebral Aneurysm Detection and Analysis challenge, CADA), our proposed approach achieved superior performance with a Dice similarity coefficient (DSC) score of 0.8967, which is 11.56% higher than the classical 3D U-Net (DSC: 0.8967 vs. 0.8038) and 1.20% higher than the current best results reported by Ma et al. [29] (DSC: 0.8967 vs. 0.8861), demonstrating a clear advancement over existing baseline methods in IAs segmentation. Furthermore, when compared to state-of-the-art 3D brain tumor segmentation approaches on the BraTS 2020 MRI dataset, our proposed method obtained the highest segmentation performance with a mean DSC of 0.8535, indicating its robustness and generalizability.

## 2. Materials and Methods

### 2.1. Overall Framework

**Figure 1:**
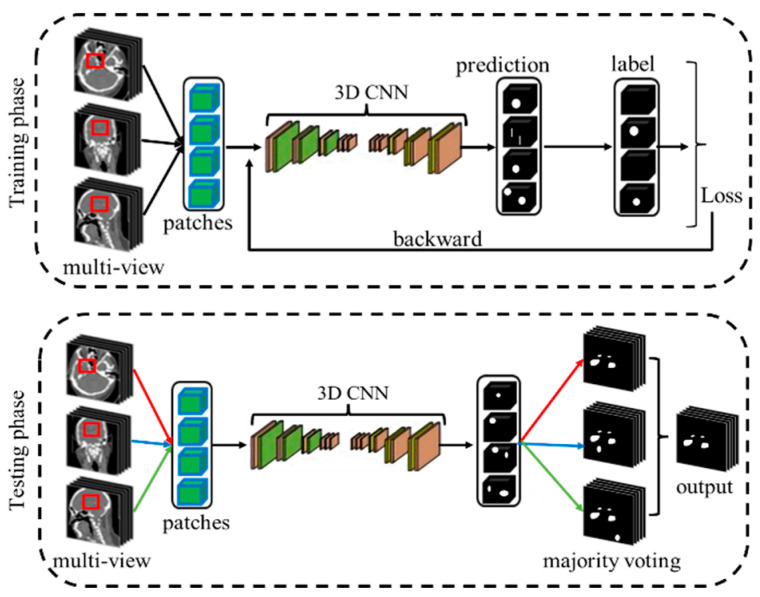
The overall flow of the proposed method for IAs segmentation.

**Figure 2:**
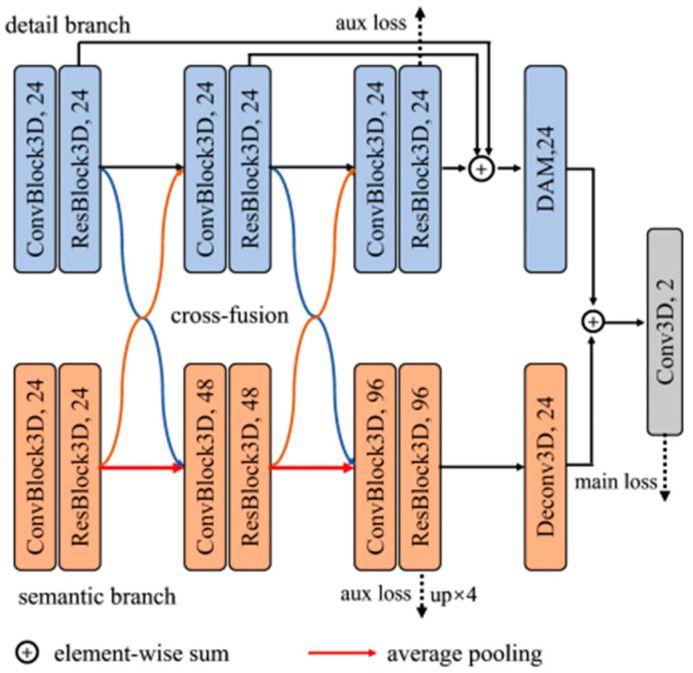
The architecture of the proposed DPF-Net.

In this study, we introduce an innovative framework for the automated segmentation of IAs, as outlined in Figure 1. This framework incorporates a multi-view fusion strategy, which integrates and processes features from axial, sagittal, and coronal planes to capture a comprehensive representation of complex anatomical structures. Specifically, during the training phase, we generate patches from diverse perspectives and employ a randomized selection process for their integration into our deep learning network. This approach aims to enhance the network’s capability to depict the intricate details of IAs. In the testing phase, we employ an ensemble method that combines the segmentation results from all three views using majority voting. The pivotal component of our segmentation system is the proposed dual-path fully convolutional network, equipped with an ingeniously designed cross-fusion module and a detail aggregation module (Figure 2).

### 2.2. Network Architecture

#### 2.2.1. Dual-Path Fusion Network (DPF-Net)

As depicted in Figure 2, our novel DPF-Net is meticulously designed with two synergistic branches: a semantic branch for extracting deep, meaningful features and a detail branch dedicated to preserving intricate details. The semantic branch consists of three stages, each with three convolutional blocks, featuring an arrangement of 3 × 3 × 3 convolutional layers, instance normalization (IN), and ReLU activation. Notably, IN helps mitigate the impact of contrast variations across different samples and remains stable in small-batch settings. Therefore, we employ IN in our network to enhance feature normalization at the instance level, ensuring more consistent feature representation and improved segmentation performance. Embracing the principles of deep residual learning, identity connections are incorporated into the latter two convolutional blocks to fortify feature extraction. To down-sample the features, a 2 × 2 × 2 average-pooling layer with a stride of 2 is applied between stages. The detail branch parallels the semantic branch but uniquely omits down-sampling to maintain the finer details. Cross-fusion modules effectively bridge the rich semantic features and finer details between these two branches (detailed in Section 2.2.2). Additionally, a detail aggregation module (detailed in Section 2.2.3) integrates multi-level features from the detail branch. In the decoder phase, we upscale the semantic branch features to the input image size, combining them with the detail branch features via element-wise summation. This approach reduces the computational complexity and avoids additional computational burden. The combined features are then processed through a convolutional layer with 3 × 3 × 3 kernels to produce the final segmentation maps.

The proposed network architecture includes one main loss layer and two auxiliary loss layers strategically positioned in the final stages of both the semantic and detail branches. In the semantic branch, deconvolution layers are employed to upscale the resolution of feature maps, followed by a convolutional layer with 3 × 3 × 3 kernels to generate auxiliary prediction maps. Similarly, in the detail branch, an auxiliary loss layer comprising a 3 × 3 × 3 convolutional layer is utilized to produce detailed prediction maps.

#### 2.2.2. Cross-Fusion Module (CFM)

**Figure 3:**
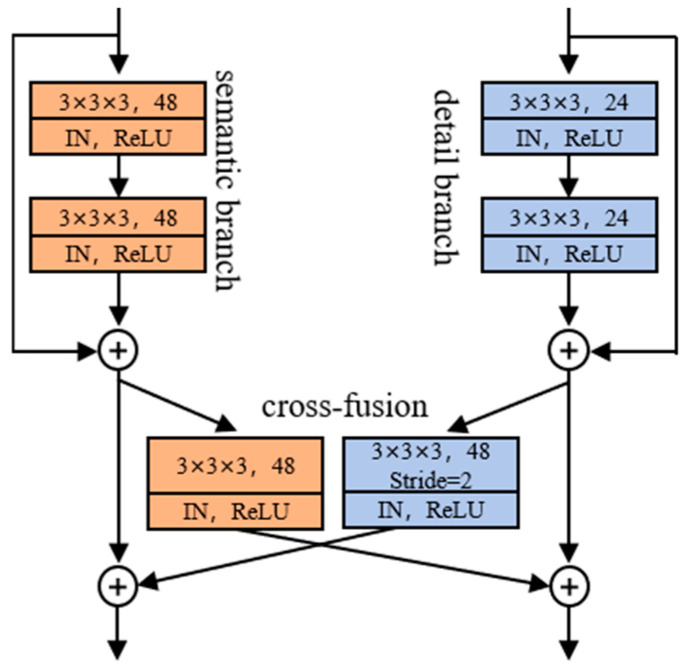
The detailed illustration of the cross-fusion module.

Inspired by DDR-Nets [30], we developed a CFM to seamlessly integrate the feature connections between the semantic and detail branches. As illustrated in Figure 3, the CFM first compresses the features from the semantic branch using a 3 × 3 × 3 convolutional block, followed by upscaling through a deconvolution block. Concurrently, features from the detail branch undergo expansion and down-sampling via 3 × 3 × 3 convolutional blocks with a stride of 2. Notably, the utilization of 3 × 3 × 3 convolutional blocks in CFM serves dual purposes: firstly, it facilitates channel adjustment, and secondly, it mitigates the potential semantic gap between the semantic and detail branches, thereby promoting effective feature fusion.

For the *i*-th feature maps $X_{s,i}$ from the semantic branch and $X_{d,i}$ from the detail branch, the above fusion operation could be written as follows:

(1) $$\begin{array}{c} X_{s,i}=X_{s,i-1}+deconv(conv\left(X_{d,i-1}\right))\\ X_{d,i}=X_{d,i-1}+conv\left(X_{d,i-1}\right) \end{array}$$

where $conv\left(\cdot \right)$ and $deconv\left(\cdot \right)$ correspond to the 3D convolutional blocks and 3D deconvolution blocks, respectively.

#### 2.2.3. Detail Aggregation Module (DAM)

**Figure 4:**
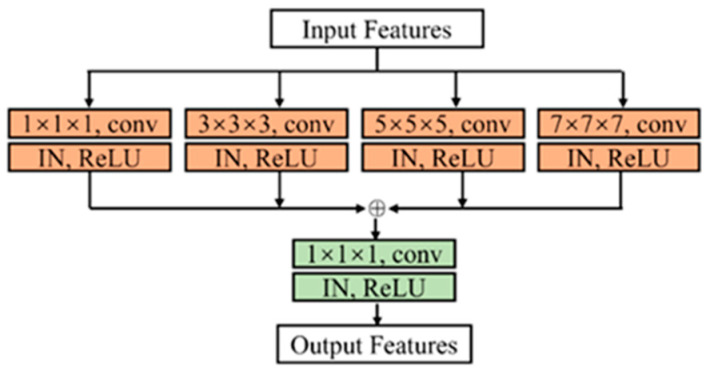
The detailed illustration of the detail aggregation module.

To further mitigate the loss of detail in the feature extraction process, we propose the DAM to integrate detail features from various pyramid levels into the detail branch. As illustrated in Figure 4, the DAM comprises four sub-branches, and each sub-branch includes a $k_{i}\times k_{i}\times k_{i}$ convolutional block for local feature extraction, we set $k_{i}$ $\left(i\in \left\{1,2,3,4\right\}\right)$ to 1, 3, 5, 7. The outputs from the four sub-branches are then combined through an element-wise sum operation, followed by a 1×1×1 convolutional block. This design enables the DAM to capture a broad spectrum of semantic contexts, thereby addressing the semantic gap between the semantic and detail branches. For the input feature map x*,* the final output of our DAM $y_{dam}$ could be written as follows:

(2) $$y_{dam}={conv}_{1}\left(\sum_{1}^{4}{conv}_{2i-1}\left(x\right)\;\right)$$

where ${conv}_{i}$ denote a i×i×i 3D convolutional block.

### 2.3. View Fusion for Intracranial Aneurysms (IAs) Segmentation

View fusion strategies, prevalently employed in 2D CNN-based methods to augment spatial feature extraction, have also shown potential in 3D CNNs. Although 3D convolution operations are typically believed to effectively extract spatial features from medical volumes, recent insights from Wang et al. [31] suggest that training models solely on a single view, such as the axial view, may be suboptimal. This is particularly true for complex objects whose characteristics might not be adequately captured from a single perspective. Furthermore, we observed that in the clinical practice of aneurysm annotation, radiologists often rely on multiple views (axial, coronal, and sagittal views) to achieve a more accurate mask. Drawing on this practical insight, we incorporated a view fusion strategy into our IAs segmentation task, aiming to harness a more comprehensive representation of the aneurysms.

We implemented dimension permutation operations to generate multi-view volumes: axial volume $X^{A}\in R^{d\times h\times w}$, coronal volume $X^{C}\in R^{h\times d\times w}$ and sagittal volume $X^{S}\in R^{w\times h\times d}$. We then created multi-view patches from these volumes using a sliding window approach. In contrast, we adopted a randomized selection strategy for these patches, feeding them into the network during the model’s optimization process. The above multi-view training approach can be summarized as follows:

(3) $$\hat{y}=f\left(x_{v,i},\;\theta \right)$$

where $x_{v,i}$ represents the patch volumes of size i×i×i $v\in \left\{A,C,S\right\}$, A, C, and S denote the axial, coronal, and sagittal planes, respectively, and θ indicates the parameters of the trained model.

### 2.4. Loss Function

IAs typically constitute only a small fraction of the total volume in medical imaging, leading to a significant foreground–background imbalance. This imbalance can pose challenges in the training phase, such as overfitting and model degradation. To counter these issues, we incorporated the Dice loss into our model’s optimization process. The Dice loss function is specifically designed to minimize the dissimilarity between the predicted results $\hat{y}$ and the ground truth y, and it is mathematically expressed as follows:

(4) $$DL\left(\hat{y},y\right)=1-\frac{2\times ∥y\cap \hat{y}∥}{∥y∥+∥\hat{y}∥}$$

To better learn the representation of different branches, the proposed model adopts a deep supervision strategy by adding auxiliary layers in the stages of the semantic and detail branches. Therefore, the loss function for the final optimization was formulated as follows:

(5) $$loss=DL\left({\hat{y}}_{main},y\right)+\beta_{1}\times DL\left({\hat{y}}_{amb},y\right)+\beta_{2}\times DL({\hat{y}}_{adb},y)$$

where $DL\left({\hat{y}}_{main},y\right)$ denotes the main loss; $DL\left({\hat{y}}_{amb},y\right)$ and $DL({\hat{y}}_{adb},y)$ denote the auxiliary loss of the semantic and detail branches, respectively; $\beta_{1}$ and $\beta_{2}$ are the balance weights used to adjust the balance between the loss, usually set to 1.

### 2.5. Implementation Details

Experiments were conducted in the open-source framework TensorFlow 1.13.1 on a computer equipped with an 11 GB GeForce RTX 2080 Ti GPU, a GenIntel(R) Core (TM) i9 13900KF 3.00 GHz CPU, 500 GB storage, and 48 GB random access memory (RAM). The Adam algorithm was adopted for model optimization, with a momentum of 0.9. The learning rate was initialized at 0.0001 and decayed by multiplying $(1-\frac{iterations}{total\;iterations}{)}^{0.9}$ as the iteration increased. In this paper, we conducted experiments with a range of patch sizes and batch sizes to investigate their effects on IAs segmentation. The most effective combination was selected for model training and prediction, with a patch size set to 64 × 64 × 64 and an input batch size of 4 (as detailed in Section 3.5). The maximum training epoch number was set to 100, with a total training time of 6.78 h. As in [4], in this study, we implemented five-fold cross-validation to evaluate the effectiveness of the proposed method. Four folds were allocated for training, with the remaining fold reserved for inference. In the pre-processing phase, the image intensity values were truncated between 1000 and 2500 to eliminate irrelevant details. Data augmentation techniques such as random flipping, random rotation, random intensity adjustments, and random introduction of Gaussian noise were utilized to enhance the model’s robustness. For post-processing, we employed a connected component analysis method to filter out potential noise in the prediction results. Specifically, connected domains smaller than 10 pixels were excluded from consideration. Statistical significance was set at *p*-value < 0.05, and all statistical analyses were performed with SPSS (26.0 for Windows).

## 3. Results

### 3.1. Experimental Dataset

CADA (Cerebral Aneurysm Detection and Analysis Challenge), which contains 109 digital subtraction angiography (DSA) cases with 127 annotated IAs, was used to evaluate the performance of the proposed method. Images were acquired utilizing the digital subtraction AXIOM Artis C-arm system using a rotational acquisition time of 5 s with 126 frames (190° or 1.5° per frame, 1024 × 1024-pixel matrix, 126 frames). Post-processing was performed using LEONARDO InSpace 3D (Siemens Healthineers, Siemensstraße 3, Forchheim, Germany). The images are 256 × 256 × 220, and slice thickness is 0.5 mm.

### 3.2. Evaluation Metrics

Four evaluation metrics were utilized to quantitatively evaluate the performance of the proposed IAs segmentation methods on the CADA dataset. As in, the metrics were calculated based on the segmentation results of complete scans rather than patches.

The Dice similarity coefficient (DSC) was used to evaluate the similarity between the predictions $\hat{y}$ and the ground truth y.

(6) $$DSC=\frac{2\times ∥y\cap \hat{y}∥}{∥y∥+∥\hat{y}∥}$$

Precision was used to compute the proportion of true-positive predictions among the true predictions.

(7) $$Precision=\frac{∥y\cap \hat{y}∥}{∥\hat{y}∥}$$

Recall was used to calculate the proportion of true-positive predictions among the ground truth.

(8) $$Recall=\frac{∥y\cap \hat{y}∥}{∥y∥}$$

The Intersection over union (IoU) was used to calculate the proportion of truth-positive predictions among the union of predictions $\hat{y}$ and ground truth y.

(9) $$IoU=\frac{∥y\cap \hat{y}∥}{∥y\cup \hat{y}∥}$$

The DSC, Precision, Recall, and IoU values are scaled from 0 to 1, with larger values indicating more accurate segmentation performance. Additionally, parameter size (Param.) is recorded to assess network complexity.

### 3.3. Comparison with Other Methods on the CADA Dataset

**Figure 5:**
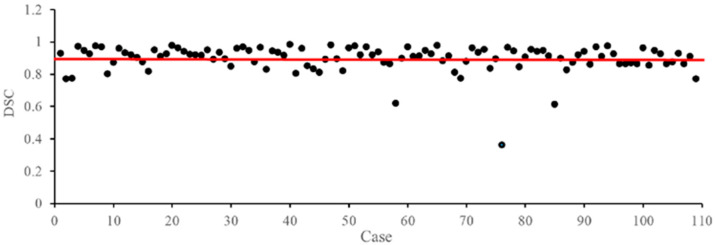
DSC score obtained by applying our proposed method to each volume in the CADA dataset. The red line indicates the mean of all cases.

To evaluate the effectiveness of our proposed method, we conducted a comparative analysis with seven existing IAs segmentation methods. The first five quantitative results in Table 1 were either directly obtained from the original authors or extracted from published papers, and the last two (i.e., V-Net and SwinUNetR) were trained from scratch as our proposed DPF-Net. Specifically, the performance metrics for U-Net, Attention U-Net, and Trans U-Net were sourced from [4]. As detailed in Table 1, our method outperformed these existing approaches, achieving a mean DSC of 0.8967, a mean Precision of 0.9033, and a mean IoU of 0.8214. Notably, while the mean Recall reported in [29] was slightly higher than ours, our method outshined in both the mean DSC (0.8967 vs. 0.8861) and Precision (0.9033 vs. 0.8934). Figure 5 illustrates the mean DSC values across all test cases and reaffirms our method’s consistency and robustness, with only a minority of cases falling below the 70% threshold. These findings underscore our method’s proficiency in delivering both satisfactory and stable results on the CADA dataset.

To further validate the robustness of our proposed method, we evaluated its performance across different IA size conditions. Based on the IAs diameter, cases were classified into three groups: Small (r < 5 mm), Medium (5 ≤ r < 15 mm), and Large (r ≥ 15 mm). As shown in Table 2, all methods achieved higher segmentation accuracy in the Large group compared to the Small and Medium groups, primarily because larger aneurysms exhibit more distinct features, making them easier to identify. Furthermore, our method outperformed other approaches (e.g., V-Net and SwinUNetR) in both the Small and Medium groups. Notably, in the Small group, our method achieved the highest segmentation performance, surpassing SwinUNetR by 39.32% (DSC: 0.8616 vs. 0.6184) and V-Net by 51.04% (DSC: 0.8616 vs. 0.5704), demonstrating its superior capability in accurately segmenting small IAs.

**Figure 6:**
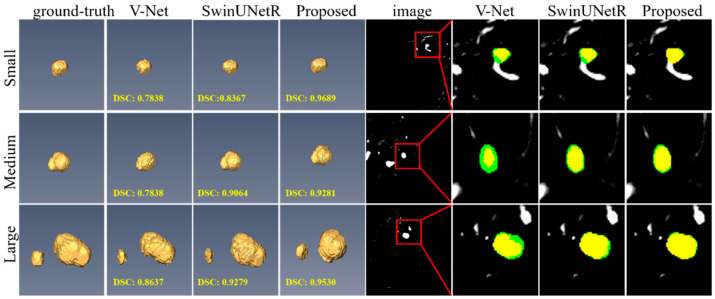
Segmentation performance of the proposed method on the CADA dataset. The ground truth and prediction results are marked in green and red, respectively. Yellow regions represent the overlap of prediction and ground truth. Note the images are not on the same scale.

Figure 6 presents the qualitative results of our approach. Despite the substantial variations in the sizes, locations, shapes, and numbers of IAs across the DSA volumes, our method demonstrated a closer alignment with manual delineations compared to other methods, such as V-Net and SwinUNetR. This is particularly evident for smaller aneurysms, as shown in the first row of Figure 6. The observed improvement can primarily be attributed to the network’s efficient utilization of fine-grained features.

### 3.4. Ablation Studies

We explored the impact of individual components on the performance of our proposed segmentation model through ablation studies by employing the full model, network 1, as a baseline. We systematically evaluated the following variants: network 2, excluding the detail branch; network 3, without the DAM; and network 4, lacking the CFM. The segmentation outcomes of these configurations, detailed in Table 3, revealed the significance of each module in enhancing model accuracy.

#### 3.4.1. Comparison with the Model Without Detail Branch

**Figure 7:**
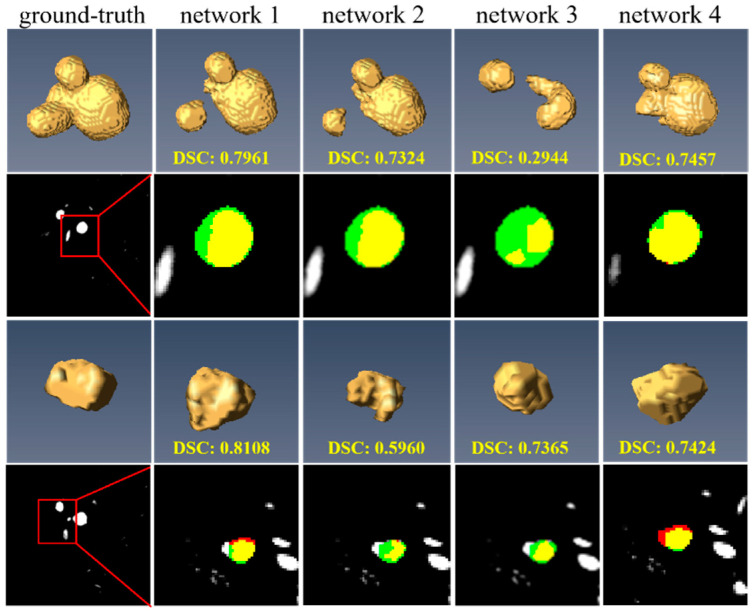
The segmentation results of ablation studies on the CADA dataset. The ground truth and prediction results are marked in green and red, respectively. Yellow regions represent the overlap of prediction and ground truth. Network 1 (full proposed model), network 2 (without detail branch), network 3 (without detail aggregation module), and network 4 (without cross-fusion module). Note the images are not the same.

The efficacy of the detail branch is evident in Table 3. Specifically, network 1, which incorporates the detail branch, achieved a mean DSC of 0.901, a Recall of 0.907, a mean Precision of 0.907, and a mean IoU of 0.825. These results reflect improvements over network 2 (without the detail branch), with improvements of 5.03% in DSC, 3.01% in Recall, 3.80% in Precision, and 7.71% in IoU. Notably, as depicted in Figure 7, this enhancement is particularly pronounced in the detection of smaller IAs. These results underscore the detail branch’s critical contribution to the model’s overall accuracy, highlighting its potential in detecting smaller pathological features.

#### 3.4.2. Comparison with the Model Without Detail Aggregation Module

To evaluate the effectiveness of DAM, we compared the segmentation results of network 1 (with DAM) and network 3 (without DAM). As shown in Table 3, network 3 demonstrated lower performance metrics (DSC (0.866), Recall (0.872), and IoU (0.786)) than network 1. As indicated in Figure 7, network 3 faces a more serious underestimation issue in segmenting IAs than network 1, as illustrated in column 4 of Figure 7. This issue is primarily attributed to the insufficient capture of detailed features. DAM, as shown in Figure 4, effectively harnesses rich low-level details through a convolutional pyramid block, leading to improved segmentation performance in network 1 compared to network 3.

#### 3.4.3. Comparison with the Model Without Cross-Fusion Module

To investigate the contribution of CFM, we compared network 1 (with CFM) with network 4 (without CFM). Table 3 shows that network 1 outperformed network 4 in terms of DSC (0.9007 vs. 0.8913), Precision (0.9072 vs. 0.8748), and IoU (0.8245 vs. 0.8087). Contrastingly, network 4 achieved a higher Recall (0.9220), indicating a greater propensity for over-segmentation, as depicted in column 5 of Figure 7. These results underscore the necessity of harmonizing detailed and high-level semantic features to curtail false positives and enhance segmentation precision.

### 3.5. Impact of Patch Size and Batch Size

The interplay between patch size and batch size is pivotal in optimizing segmentation models for IAs. Larger patches, while informative, skew the model towards foreground-background imbalance, detrimentally affecting the detection of small aneurysms. Smaller patches, focusing on finer features, risk under-segmentation due to insufficient contextual information. As detailed in Table 4, we evaluated various patches (48 × 48 × 48, 64 × 64 × 64, 96 × 96 × 96) and batch sizes under GPU memory constraints. The combination of a 64 × 64 × 64 patch size with a batch size of 4 emerged as optimal. In contrast, larger patches (96 × 96 × 96) led to pronounced under-segmentation issues, particularly for smaller aneurysms. Additionally, increased batch sizes were found to extend training times. Consequently, a patch size of 64 × 64 × 64 and a batch size of 4 were adopted for their efficacy and efficiency.

### 3.6. Impact of Deep Supervision

The deployment of deep supervision, a pivotal training approach characterized by the integration of an auxiliary loss layer, was examined for its influence on model performance in segmentation tasks. Comparative analyses, as elucidated in Table 5, revealed that networks harnessing deep supervision surpassed those without by 1.79% in DSC, 0.63% in Recall, 1.82% in Precision, and 2.73% in Intersection over Union (IoU). These performance improvements underscore the efficacy of deep supervision in refining the model’s feature extraction capabilities, thereby enhancing overall segmentation precision.

### 3.7. Impact of Different View Fusion Manners

**Figure 8:**
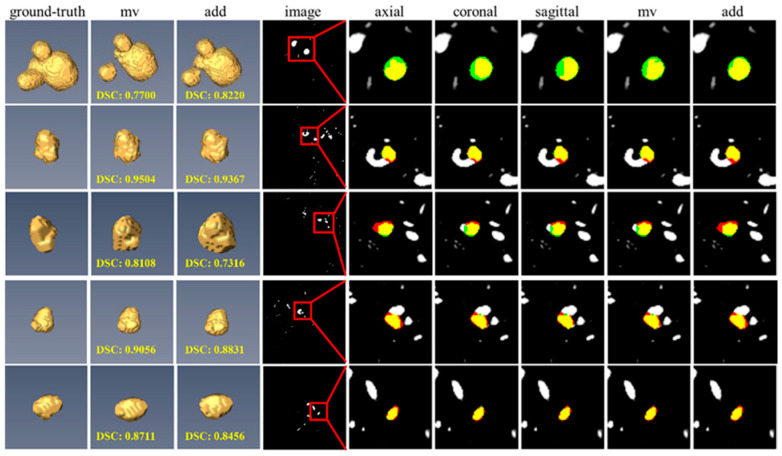
Segmentation performances of view fusion when using different fusion manners. The axial, coronal, and sagittal indicate prediction results obtained from axial, coronal, and sagittal views, respectively. The ground truth and prediction results are marked in green and red, respectively. Yellow regions represent the overlap of prediction and ground truth. The abbreviations mv and add denote fusing multi-view pre-diction results via majority voting and adding operation, respectively.

In the testing phase, two predominant view fusion methodologies (i.e., summation and majority voting) were explored. As shown in Figure 8, the summation operation could effectively utilize multi-view predictions and alleviate the under-segmentation problems (Figure 8, line 1) but suffer from the over-segmentation challenges (Figure 8, line 3). The majority voting operation could effectively improve the stability of final segmentation results and avoid extreme results (e.g., miss-segmentation) but endure suboptimal problems (Figure 8, line 1). Table 6 demonstrates that the majority voting technique marginally outperforms the summation approach. Consequently, the final segmentation results are derived from multi-view predictions using the majority voting method.

### 3.8. Failure Case Analysis on the CADA Dataset

**Figure 9:**
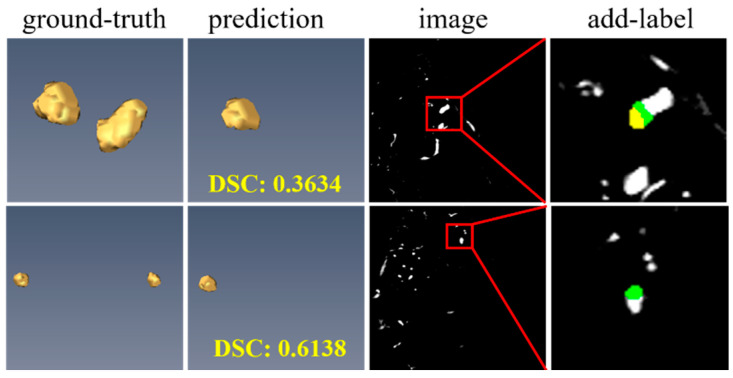
Visualization of failure cases on the CADA dataset. The ground truth and prediction results are marked in green and red, respectively. Yellow regions represent the overlap of prediction and ground truth.

Despite the superior segmentation performance of our method compared to other approaches, our extensive experimental analysis reveals certain cases where the segmentation results are not fully satisfactory, as illustrated in Figure 9. These cases suggest that our method may fail to properly segment small IAs regions when multiple IAs instances are present in the same volume, leading to under-segmentation. A potential reason for this is that our segmentation system has not effectively balanced the pixel-level information of the target with the instance-level information during training, resulting in insufficient attention given to smaller instances. An improved optimization strategy that better integrates pixel and instance-level constraints may help address this issue.

### 3.9. Evaluation of the Brain Tumor Segmentation Tasks

**Figure 10:**
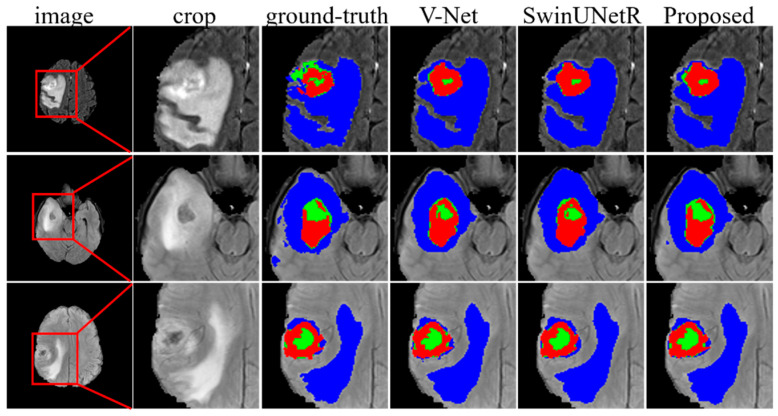
Segmentation performance on BraTS 2020 brain tumor segmentation dataset. WT is marked in blue, green, and red; TC is marked in green and red; ET is marked in red.

To demonstrate the robustness and generalizability of our proposed method, we conducted additional experiments on the BraTS 2020 MRI dataset [37,38], which is widely used for brain tumor segmentation. The BraTS 2020 dataset includes 369 annotated MRI scans for model training. Each scan includes four aligned modalities (FLAIR, T1, T1ce, and T2) along with their corresponding labels, which contain four classes: background, NCR/NET, ED, and ET. Assessment is based on three different brain tumor regions: Whole Tumor (WT = NCR/NET + ED + ET), Tumor Core (TC = NCR/NET + ET), and Enhancing Tumor (ET). Five-fold cross-validation is used to evaluate the effectiveness of our proposed method. As shown in Table 7, our method outperformed others, achieving a mean DSC of 0.8535 and individual DSC scores of 0.9058, 0.8528, and 0.8002 for WT, TC, and ET, respectively. Additionally, as shown in Figure 10, our method showed a closer alignment with manual delineations, particularly for the relatively small ET regions. This performance improvement can be attributed to the ability of DPF-Net to preserve fine details, enhancing the network’s sensitivity to small targets and resulting in better segmentation performance.

## 4. Discussion

### 4.1. A New Framework for Preserving Detail Features in IAs Segmentation

In clinical practice, the accurate segmentation of IAs is of great significance for understanding patient conditions and assisting clinicians in formulating effective therapeutic strategies. Currently, IAs segmentation largely depends on manual delineation. This method is unsuitable for the pressures of the rapid response in aneurysm rupture scenarios and the escalating demands of diagnosis. To overcome the limitations of manual delineation, numerous deep learning-based methods have been developed. These approaches typically focus on enhancing semantic information to improve segmentation performance. However, they tend to overlook the critical role of detailed information in IAs segmentation. This oversight is critical, especially because IAs are small, and their distinctive features may be lost during down-sampling in semantic feature extraction, potentially leading to missed aneurysms. Our study addresses this issue by preserving detailed information. We introduced a detail branch that maintains image feature resolution to mitigate the loss of aneurysm details during down-sampling. Recognizing the importance of both detail and semantic information in segmentation, we incorporated a cross-fusion module in the encoder to facilitate the interaction of these two types of information, thereby enhancing the network’s ability to extract IAs features. Furthermore, we designed a detail aggregation module to integrate information from various stages of the detail branch using convolutional pyramid operations. This approach effectively bridges the gap between finer detail and richer semantic features, leading to a more cohesive integration in the decoder phase.

Compared to advanced models, our proposed DPF-Net achieved the best segmentation performance on the CADA dataset, with a mean DSC score of 0.8967. This exciting result leads us to believe that our study will significantly contribute to advancing the incorporation of detail features in network design for IAs segmentation. The superior segmentation performance on the BraTS 2020 MRI brain tumor dataset further validates the robustness and adaptability of our proposed method. This suggests that our approach can be effectively transferred to other imaging modalities (e.g., computed tomographic angiography, CTA) for various segmentation tasks, broadening its potential medical applications. Additionally, given the faster inference speed (DPF-Net: 43 s/case, V-Net: 47 s/case, SwinUNetR: 52 s/case) and smaller parameter size (DPF-Net: 1.45 MB, V-Net: 3.75 MB, SwinUNetR: 16.83 MB) of our proposed model (Table 1), developing a user-friendly interface platform based on our approach would be highly beneficial in assisting manual annotation. Such a platform has the potential to significantly enhance annotation efficiency and accuracy, ultimately contributing to improved surgical planning and postoperative monitoring.

### 4.2. Integrity of Spatial Structures Is Important for IAs Segmentation

Due to the small size of IAs, 3D networks commonly adopt a patch-based approach to address the foreground-background imbalance. However, the diverse anatomical locations of IAs make prior-based patch generation challenging, leading to the widespread use of sliding window sampling. While straightforward, this method disrupts spatial integrity, limiting feature learning and often causing under-segmentation. Although mask-based patch generation during training helps preserve IAs structures, the testing phase still relies on sliding window sampling, perpetuating incomplete spatial representation. To address this, we introduce a view fusion strategy that enhances IAs representation through multi-perspective sampling. While it alleviates spatial disruptions, it does not fully resolve them. Maintaining spatial integrity in patch-based segmentation remains a critical challenge requiring further research and refinement.

### 4.3. Limitations and Future Work

Although our proposed model has shown promise on the CADA and BraTS 2020 datasets, there are still some limitations. First, the proposed method mainly focuses on the overall performance of IAs, ignoring the exploration of some challenging cases, which are prone to over- or under-segmentation issues. Second, although our initial validation using the established CADA dataset demonstrates the proposed method’s effectiveness, broader investigations across various modalities (e.g., CTA) remain essential. Third, while our method reduces model complexity by introducing a detail branch to bypass cumbersome step-by-step up-sampling in the encoder, it still relies on traditional convolutions, leading to a significant computational burden. This hinders the segmentation of larger targets (e.g., liver, brain), which require larger patch sizes to capture their overall structure.

In the future, we will pay more attention to the extraction of these cases by using hard example mining methods (e.g., the subset selection strategy and online hard example mining) to enhance the contribution of the hard samples in the training phase to further enhance the segmentation performance of the proposed method. Furthermore, we plan to validate our method on datasets from various centers and modalities to thoroughly assess its effectiveness. Moreover, we aim to replace traditional convolutions in our DPF-Net with lightweight depth-wise separable convolutions to facilitate model deployment and broader application.

## 5. Conclusions

In this paper, we proposed a dual-path fusion network for efficient IAs segmentation, incorporating a detail branch to preserve fine-grained features, along with a cross-fusion module and a detail aggregation module to enhance contextual information interaction. Additionally, a view fusion strategy is employed to enrich feature representation. Experimental results on publicly available CADA and BraTS 2020 datasets demonstrated the effectiveness of the proposed method, while ablation studies further verified the contributions of the proposed modules to performance improvements. In the future, more advanced feature representation architectures and modules will be explored to enhance the model’s ability to efficiently capture features in target regions. Moreover, the integration of clinical information will be leveraged to improve the structural accuracy of the segmentation outcomes.

## Figures and Tables

**Table 1:** Quantitative comparison of the proposed model with state-of-the-art methods on the CADA dataset. The abbreviation IS denotes inference speed (second/case).

Methods	DSC	Recall	Precision	IoU	*p*-Value	IS	Param. MB
U-Net [32]	0.8038 ± 0.0358	0.8503 ± 0.0528	0.7921 ± 0.0547	0.6978 ± 0.0413	-	-	
Attention U-Net [33]	0.8207 ± 0.0341	0.8224 ± 0.0447	0.8439 ± 0.0147	0.7197 ± 0.0379	-	-	
Trans U-Net [34]	0.8311 ± 0.0250	0.8339 ± 0.0446	0.8518 ± 0.0280	0.7344 ± 0.0299	-	-	
Ou et al. [4]	0.8368 ± 0.0242	0.8587 ± 0.0357	0.8397 ± 0.0327	0.7381 ± 0.0293	-	-	
Ma et al. [29]	0.8861	**0.9036**	0.8934	-	-	-	
V-Net [35]	0.8137 ± 0.1779	0.8469 ± 0.1974	0.8039 ± 0.1537	0.7027 ± 0.1785	<0.001 ^a^	47	3.75
SwinUNetR [36]	0.8498 ± 0.1485	0.8638 ± 0.1458	0.8375 ± 0.1397	0.7845 ± 0.1473	<0.001 ^a^	52	16.83
Proposed	**0.8967 ± 0.0837**	0.8972 ± 0.1199	**0.9033 ± 0.1084**	**0.8214 ± 0.1164**	**-**	43	1.45

The highest results are shown in bold. ^a^ Pairwise comparisons revealed significant differences between the proposed method and the other methods (i.e., V-Net, SwinUNetR) across all performance metrics (i.e., DSC, Recall, Precision, and IoU).

**Table 2:** Quantitative comparison under different IA sizes conditions in terms of DSC. *N* means the number of cases.

Methods	Small (*N* = 15)	Medium (*N* = 88)	Large (*N* = 6)	*p*-Value
V-Net [35]	0.5704 ± 0.2327	0.8478 ± 0.1539	0.9217 ± 0.0872	<0.001 ^a^
SwinNetR [36]	0.6184 ± 0.1869	0.8539 ± 0.1275	**0.9317 ± 0.0735**	<0.001 ^a^
Proposed	**0.8616 ± 0.0816**	**0.9005 ± 0.0840**	0.9281 ± 0.0686	

The highest results are shown in bold. ^a^ Pairwise comparisons revealed significant differences between the proposed method and the other methods (i.e., V-Net, SwinUNetR) in Small and Medium IAs groups.

**Table 3:** Segmentation performance of ablation studies on the CADA dataset at one testing fold.

Methods	DSC	Recall	Precision	IoU	*p*-Value
network 1	**0.9007 ± 0.0615**	0.9072 ± 0.1009	**0.9072 ± 0.0855**	**0.8245 ± 0.1000**	**-**
network 2	0.8576 ± 0.1140	0.8807 ± 0.1544	0.8740 ± 0.1448	0.7655 ± 0.1574	<0.005 ^a^
network 3	0.8657 ± 0.1551	0.8724 ± 0.2049	0.9056 ± 0.0828	0.7863 ± 0.1813	<0.005 ^b^
network 4	0.8913 ± 0.0590	**0.9220 ± 0.0906**	0.8748 ± 0.0922	0.8087 ± 0.0950	<0.005 ^c^

The highest results are shown in bold. ^a^ Pairwise comparisons revealed significant differences between network 1 and network 2 across all performance metrics (i.e., DSC, Recall, Precision, and IoU). ^b^ Pairwise comparisons revealed significant differences between network 1 and network 3 in DSC Recall and IoU. ^c^ Pairwise comparisons revealed significant differences between network 1 and network 4 in Precision and IoU.

**Table 4:** Segmentation results on the CADA dataset using different patch and batch sizes.

Patch Size	Batch Size	DSC	Recall	Precision	IoU
48 × 48 × 48	2	0.8664 ± 0.0961	**0.9317 ± 0.0939**	0.8237 ± 0.1294	0.7754 ± 0.1403
	4	0.8625 ± 0.1407	0.8957 ± 0.1281	0.8466 ± 0.1704	0.7780 ± 0.1710
	6	0.8678 ± 0.1335	0.8899 ± 0.1402	0.8565 ± 0.1535	0.7847 ± 0.1652
	8	0.8760 ± 0.1203	0.8665 ± 0.1284	0.8666 ± 0.1284	0.7956 ± 0.1625
64 × 64 × 64	2	0.8572 ± 0.1237	0.8825 ± 0.1252	0.8581 ± 0.1690	0.7676 ± 0.1715
	4	**0.9007 ± 0.0615**	0.9072 ± 0.1009	**0.9072 ± 0.0855**	**0.8245 ± 0.1000**
	6	0.8722 ± 0.0990	0.8903 ± 0.1297	0.8670 ± 0.1153	0.7852 ± 0.1436
96 × 96 × 96	2	0.7710 ± 0.3100	0.7991 ± 0.3219	0.8643 ± 0.1547	0.6980 ± 0.3001

The highest results are shown in bold.

**Table 5:** Comparison between the proposed model and network without the deep supervision on the CADA dataset at one testing fold. The abbreviation DS denotes deep supervision.

Methods	DSC	Recall	Precision	IoU
w/DS	**0.9007 ± 0.0615**	**0.9072 ± 0.1009**	**0.9072 ± 0.0855**	**0.8245 ± 0.1000**
w/o DS	0.8849 ± 0.0848	0.9015 ± 0.1421	0.8910 ± 0.0885	0.8026 ± 0.1263

The highest results are shown in bold.

**Table 6:** Comparison between the proposed model and network without the deep supervision on the CADA dataset at one testing fold. The abbreviations mv and add denote fusing multi-view pre-diction results via majority voting and adding operation, respectively.

Methods	DSC	Recall	Precision	IoU
add	0.8927 ± 0.0662	**0.9417 ± 0.0774**	0.8608 ± 0.1098	0.8121 ± 10.46
mv	**0.9007 ± 0.0615**	0.9072 ± 0.1009	**0.9072 ± 0.0855**	**0.8245 ± 0.1000**

The highest results are shown in bold.

**Table 7:** Performance on the BraTS 2020 dataset for brain tumor segmentation in terms of DSC. Abbreviations WT, TC, and ET denote whole tumor, tumor core, and enhanced tumor, respectively.

Methods	WT	TC	ET	Mean	*p*-Value
Maheshwari et al. [39]	0.8730	0.7420	0.7530	0.7893	-
Zhang et al. [40]	0.8940	0.8250	0.7340	0.8177	-
Liu et al. [41]	0.9048	0.8522	0.7940	0.8503	-
Lefkovits et al. [42]	0.9000	0.8400	0.7800	0.8400	-
Fang et al. [43]	0.9050	**0.8850**	0.7480	0.8460	-
V-Net [35]	0.8914 ± 0.0635	0.8297 ± 0.1638	0.7693 ± 0.1933	0.8301 ± 0.1583	<0.05 ^a^
SwinUNetR [36]	0.9038 ± 0.0593	0.8538 ± 0.1579	0.7717 ± 0.1889	0.8431 ± 0.1468	<0.05 ^b^
Proposed	**0.9058 ± 0.0547**	0.8528 ± 0.1400	**0.8002 ± 0.1763**	**0.8535 ± 0.1385**	

The highest results are shown in bold. ^a^ Pairwise comparisons revealed significant differences between the proposed method and V-Net in TC and ET regions segmentation performance in terms of DSC. ^b^ Pairwise comparisons revealed significant differences between the proposed method and SwinUNetR in ET regions segmentation performance in terms of DSC.

## Data Availability

The data generated or analyzed during the study are available from the corresponding author upon reasonable request.
